# Syncope and Cannabis: hypervagotonia from chronic abuse? A case report and literature review

**DOI:** 10.1186/s12872-023-03566-4

**Published:** 2023-10-25

**Authors:** Marco Licciardi, Elena Utzeri, Maria Francesca Marchetti, Vincenzo Nissardi, Giovanni Cecchetto, Massimo Montisci, Roberta Montisci

**Affiliations:** 1 Department of Medical Sciences and Public Health, Clinical Cardiology, University of Cagliari, Cagliari, Italy; 2 Department of Cardiologic, Thoracic and Vascular Sciences and Public Health, Section of Legal Medicine, University of Padova, Padova, Italy

**Keywords:** Cannabis, Cardiovascular effects, Recurrent Syncope, Asystole, ILR implantation, Cardiac pace-maker

## Abstract

**Background:**

Cannabis is the most consumed drug worldwide and number of users is increasing, particularly among youth. Moreover, cannabis potential therapeutic properties have renewed interest to make it available as a treatment for a variety of conditions. Albeit rarely, cannabis consumption has been associated with cardiovascular diseases such as arrhythmias, myocardial infarction (MI) and potentially sudden death.

**Case presentation:**

A 24-year-old woman presented to the emergency department sent by her cardiologist because of a recent finding of a 16 seconds asystole on the implantable loop recorder (ILR) she implanted 7 months before for recurrent syncopes. She declared that she is a heavy cannabis user (at least 5 cannabis-cigarette per day, not mixed up with tobacco, for no less than 12 years) and all syncopes occurred shortly after cannabis consumption. After a collective discussion with the heart team, syncope unit, electrophysiologists and toxicologist, we decided to implant a dual chamber pacemaker with a rate response algorithm due to the high risk of trauma of the syncopal episodes. 24 months follow-up period was uneventful.

**Conclusions:**

Cannabis cardiovascular effects are not well known and, although rare, among these we find ischemic episodes, tachyarrhythmias, symptomatic sinus bradycardia, sinus arrest, ventricular asystole and possibly death. Because of cannabis growing consumption both for medical and recreational purpose, cardiovascular diseases associated with cannabis use may become more and more frequent. In the light of the poor literature, we believe that cannabis may produce opposite adverse effects depending on the duration of the habit. Acute administration increases sympathetic tone and reduces parasympathetic tone; conversely, with chronic intake an opposite effect is observed: repetitive dosing decreases sympathetic activity and increases parasympathetic activity. Clinicians should be aware of the increased risk of cardiovascular complications associated with cannabis use and should investigate its consumption especially in young patients presenting with cardiac dysrhythmias.

## Background

Cannabis is the most consumed drug worldwide, counting roughly 200 million users in 2019 (4% of the global population) [1]. Once illegal in most of the world countries, cannabis is now legal for medical and recreational use in several states. During the last 20 years, we have observed a growing decriminalization wave parallel with an increased number of consumers: it is therefore mandatory not only for the cardiologists but for every physician to be aware of cannabis potential cardiovascular adverse health effects [2]. We present a case report of cannabis induced asystole in a 24-year-old heavy cannabis consumer, focusing on the infrequently reported association between asystole and chronic cannabis use and we try to explain the underlying mechanisms against the background of the current literature.

### Case presentation

**Fig. 1:**
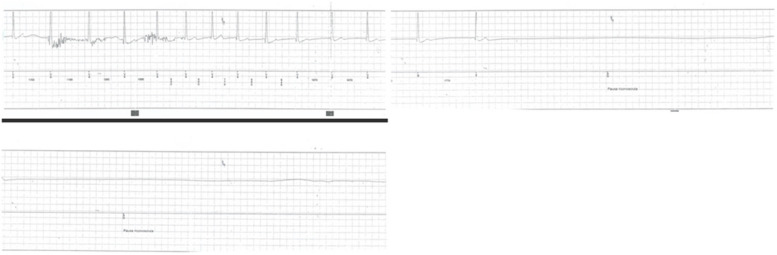
16 seconds asystole recorded by the ILR occurred around 1:00 a.m. while the patient was sitting, watching television

A 24-year-old woman presented to the emergency department sent by her cardiologist because of a recent finding of a 16 seconds asystole on the implantable loop recorder (ILR) she implanted 7 months before for recurrent syncopes. She openly declared that she was a heavy cannabis user (at least 5 cannabis-cigarette per day, not mixed up with tobacco, for no less than 12 years), confirmed by hair toxicological analyses. She had a history of at least 2 spontaneous atypical reflex syncopal episodes and a multitude of pre-syncopal episodes that began one year before admission. Her past history was remarkable for febrile seizures at age 16. The first syncopal episode was preceded by prodromes such as bilateral arms paresthesia and dyspnea. Bystanders reported tonic–clonic seizures, morsus and urinary loss followed by a relatively quick regain of consciousness. After the first syncope, because of her past history of febrile seizures, diagnostic neurological work-up aimed to exclude epilepsy, which was ruled out. After that, a 24-h dynamic electrocardiogram (ECG) was performed and revealed a I° atrio-ventricular block (AVB) and sporadic episodes of first type II° AVB. At this point, a loop recorder was implanted and tilt test was performed, revealing a positive response for hypotension preceded by classical prodromes. Under these circumstances, after 7 months from ILR implantation, a second prodrome-less syncopal episode occurred while watching television at late night and a 16 seconds asystole was found on the ILR, leading to hospitalization (Fig. 1, Table 1). Notably, both syncopal episodes occurred while she was sitting and shortly after cannabis smoking.

**Table 1:** Timeline of events that lead to hospitalization

Time	Event	Diagnostic work—up	Findings
0	First syncopal episode	EEG and brain MRI	Epilepsy exclusion
Month 1	Pre-syncopal episodes	24H dynamic ECG	First degree atrio-ventricular block (AVB); Sporadic episodes of first type second degree AVB
Month 2	Pre-syncopal episodes	Tilt test	Positive response for hypotension preceded by classical prodromes
Month 2	Pre-syncopal episodes	ILR implantation	
Month 7	Second syncopal episode		16 s asystole and admission

**Fig. 2:**
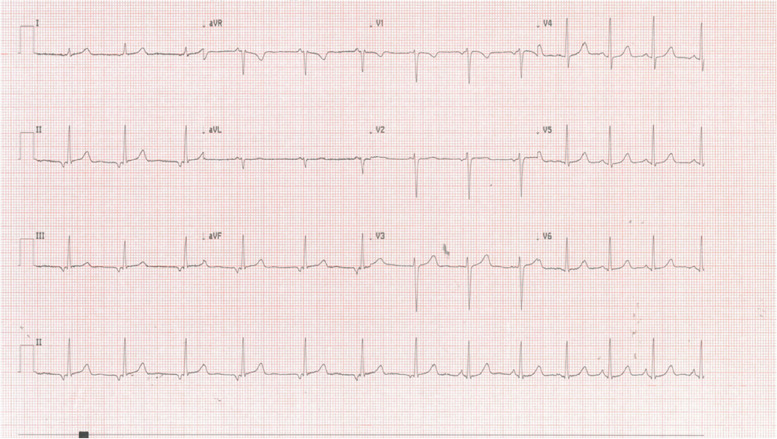
One of the several ECG showing a wandering pacemaker with a low atrial rhythm (also known as coronary sinus rhythm). We speculate that this resting ECG suggests a tonically active parasympathetic status on the conduction system of the heart

**Fig. 3:**
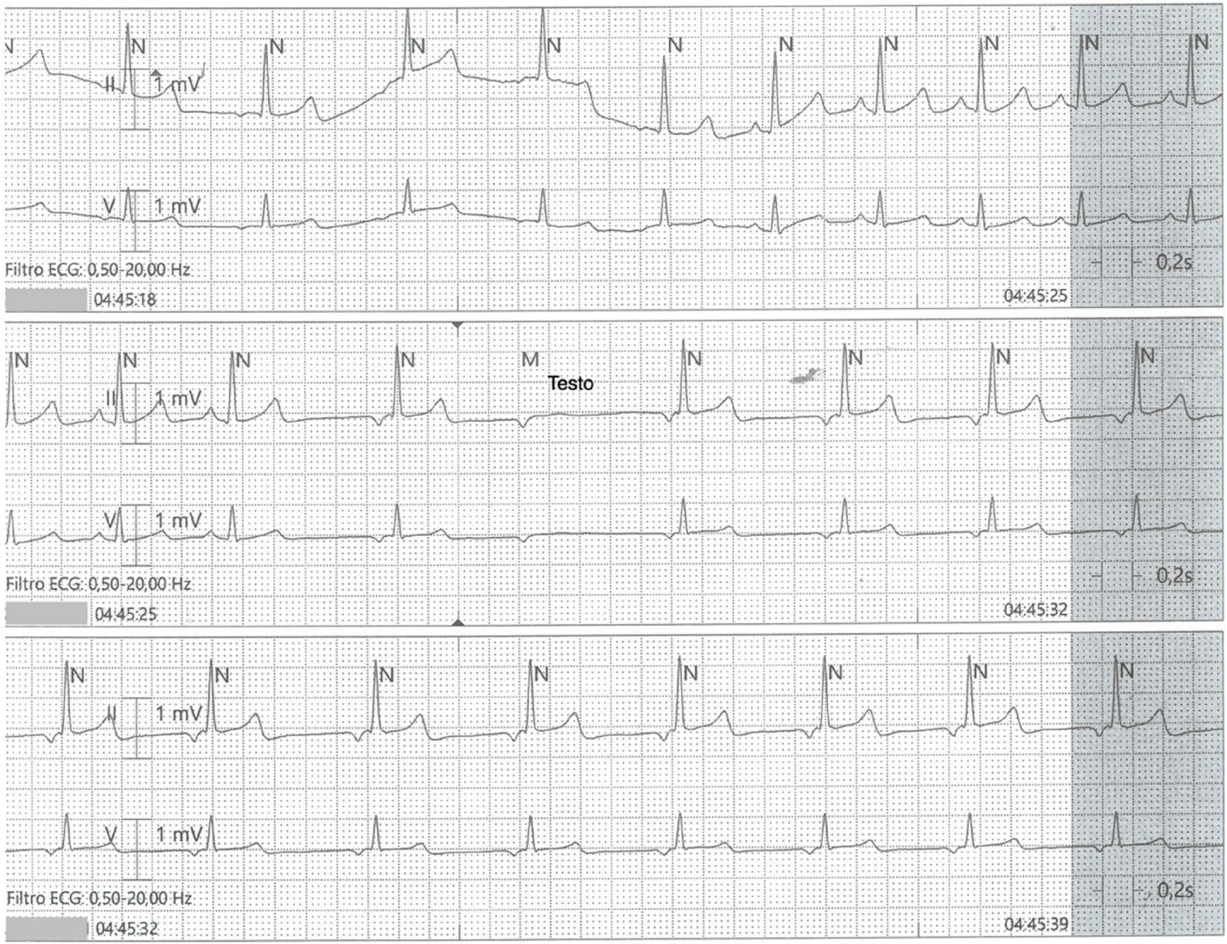
Wandering pacemaker followed by a blocked atrial wave at telemetry, occurred while the patient was sleeping (04.45 am)

On admission her vital signs were within normal limits: BP 100/70mmHg, HR 101, O2 saturation on room-temperature air was 98%. During hospitalization the patient did not experience syncopal episodes, although she reported occasional dyspnea while resting, dizziness and palpitations. However, none of these symptoms was related to remarkable changes in hearth rhythm, heart rate, O2 saturation and both sitting and standing blood pressure. The echocardiographic findings were normal. Serial ECGs showed wandering pacemaker (Fig. 2), which was also revealed at 24h telemetry, usually preceded by sinus tachycardia (Fig. 3). ILR was interrogated every time she referred palpitations, but no significant findings were detected. Given the age of our patients, her history, and clinical findings, we considered a heart conduction system anomaly unlikely. Eventually, the patient underwent a toxicological and a psychiatric evaluation, where she strongly expressed not wanting to abandon cannabis abuse providing a written informed consent. After a collective discussion with the heart team, syncope unit, electrophysiologists and toxicologist, we decided to implant a dual chamber pacemaker, due to the high risk of trauma of the syncopal episodes. 24 months follow-up period was uneventful, apart from sporadic pre-syncopal episodes (Table 2). In fact, the patient became pregnant 15 months after pacemaker implantation and she declared she finally stopped smoking cannabis, reporting a further reduction in pre-syncopal episodes. Ventricular pacing burden at 24 months follow-up was around 14%. No tachyarrhythmia and bradyarrhythmia were ever recorded.

**Table 2:** 24 Months follow up timeline of events

Time	Event	Cannabis consumption	PM recordings
Month 6	Sporadic pre-syncopal episodes	Yes	None
Month 12	Sporadic pre-syncopal episodes; pregnancy	No (declared)	None
Month 18	Sporadic pre-syncopal episodes	No (declared)	10.7% Ventricular pacing burden
Month 24	Sporadic pre-syncopal episodes	No (declared)	14% Ventricular pacing burden: change PM settings to AAI—> DDD

## Discussion and conclusions

Herbal cannabis contains over 400 compounds, including more than 60 cannabinoids, for which the plant genus Cannabis represents their unique source. The pharmacology of most of the cannabinoids is largely unknown and the two most studied compounds are Δ9-tetrahydrocannabinol (Δ9-THC, or THC), and Cannabidiol (CBD). THC is largely responsible for the psychotropic effects of cannabis consumption, and it probably exerts its effect by interaction with two specific endogenous cannabinoid receptors known as CB1 and CB2. It is now generally accepted that these receptors are both coupled with Gi/o proteins negatively controlling cyclic adenosine monophosphate formation and Ca2 + and K + ion transport. CB1 receptors are found predominantly but not exclusively at central and peripheral nerve terminals where they mediate inhibition of transmitter release. CB2 receptors are mainly found on immune cells and tissues and, although often regarded as peripheral receptors, they have been also detected in the central nervous system. In this role, the endocannabinoid system may have important interactions with other neurotransmitters [2, 3].

Cannabis consumption is associated with numerous clinical manifestation; however, cardiovascular cannabis effects are not well known. Several clinical studies, reviews, case series and reports have highlighted an association between cannabis use and dysrhythmias, which are mainly represented by tachydysrhythmia [4]. However, association between cannabis consumption and bradycardia or other bradydysrhythmias such as sinus arrest, atrioventricular block and asystole remains limited to sporadic case reports [4–7]. Our case is an addition to the existing scant literature that reinforces the association between cannabis chronic consumption and bradydysrhythmias and syncope. In the light of the poor literature, we believe that cannabis may produce opposite adverse effects depending on the duration of the habit [8, 9]. Benowitz et al. [9] observed that initial doses of THC tended to increase supine blood pressure and heart rate. Increase in HR after acute THC administration was attenuated by pretreatment with either atropine or propranolol and nearly abolished by the combination, suggesting that THC increases heart rate by stimulating sympathetic activity and reducing parasympathetic influence on the heart. With prolonged THC administration, small but significant decreases in supine systolic and diastolic blood pressure and heart rate were observed. Administration of atropine in patients exposed to prolonged THC administration produced a greater increase in blood pressure during THC compared to before THC exposure, while administration of propranolol following atropine decreased heart rate to a greater extent before THC compared to during THC exposure. Starting from these observations, we can assume that acute administration increases sympathetic tone and reduces parasympathetic tone; conversely, with chronic intake an opposite effect is observed: repetitive dosing decreases sympathetic activity and increases parasympathetic activity. Moreover, because cannabinoids are extremely lipid soluble, they accumulate in fatty tissues, reaching peak concentrations in 4 ± 5 days. Because of the sequestration in fat, the tissue elimination half-life of THC is about 7 days. Clearly, with repeated dosage, high levels of cannabinoids can accumulate in the body and continue to exert their effect even after withdrawing from use.

Other data could be considered for explaining cannabis-induced bradydysrhythmias. Some authors described that cannabidiol (CBD) has particular tropism for adenosine receptors (probably acting as a modulator) [10, 11] and this mechanism could count for an adenosine-like effect of herbal cannabis compounds. In the light of this evidence, chronic cannabis consumption can be considered a reversible cause of bradydysrhythmias and more generally a cause of a reversible hypervagotonia.

With regards to our case, we focused on the following peculiar items of these syncopal episodes. Both episodes occurred while the patient was sitting and resting and without being preceded by palpitations; the first one was preceded by long prodromes (lasted hours) and the second one without any prodrome. Both occurred shortly after cannabis smoking. Several elements point towards a hypothetical hyper-vagotonic status: (1) spontaneous 16 s asystole preceded by a slowdown of heart rate; (2) wandering pacemaker, low atrial rhythm and blocked atrial wave at telemetry and resting ECG suggesting vagal suppression of the physiological sinus pacemaker; (3) no overt reasons that could explain increased vagal tone (the patient was not an endurance athlete). The tilt test performed with the Italian protocol resulted positive, suggesting hypotensive susceptibility and a reflex vagotonic mechanism. However, the presence of a positive vasodepressor response is not sufficient to exclude the presence of asystole during spontaneous syncope. Indeed, tilt testing could result positive in up to 47% of patients with true cardiac arrhythmic syncope [12].

Given the age of our patients, her history, and clinical findings, we considered a heart conduction system anomaly unlikely. We therefore decided to try to eliminate all possible reversible causes of bradydysrhythmias and syncope and strongly suggested our patient to stop cannabis smoking, offering her a medically supervised cannabis withdrawal after a toxicologic and a psychiatric referral. However, the patient firmly expressed not wanting to abandon cannabis abuse.

After a collective discussion, we decided to implant dual-chamber rate-modulated pacemaker (DDDR). This initial setting was chosen because of previous evidence of AV block in the 24-h dynamic electrocardiogram and to maintain AV synchrony as much as possible. We also set the rate adaptive function on (R) because of a possible incipient sinus node dysfunction. With the R function on, pacemaker was programmed to maintain atrioventricular synchrony not only at rest but also during exercise, allowing for adjustment of the pacing rate to the metabolic demands of the body.

The decision to implant the pacemaker was supported by several elements: (1) Unpredictability of the syncopal events (prodromes were not always present); (2) Ineffectiveness of non-pharmacological treatment (education, lifestyle modification and counter-pressure manoeuvres); (3) Clear correlation between syncope and ECG was established; (4) Medico-legal implications: the patient showed an impulsive behavior that could expose the patient to risk of trauma (e.g.: accidents while driving). In fact, as reflected by literature reports, we may consider cannabis as a preventable and reversible cause of both bradycardia and arrythmias in general. However, at the moment of hospitalization the patient was not considering the option of cannabis abuse withdrawal and since we had a specific temporal relationship between syncopes, ECG findings and cannabis consumption, we contemplated cardiac pacing as a potentially effective treatment option for our case, also considering that non-pharmacological maneuvers were not effective. Moreover, as previously said, we considered a heart conduction system anomaly unlikely, but we could not completely exclude it. We speculate that cannabis could have acted as concurrent cause of conduction anomaly upon a vulnerable and predisposed organism, perhaps anticipating what could have happened in the future. Nonetheless, electrophysiological study was deemed unnecessary for the decision-making process for choosing proper treatment strategy, given the clear correlation between ECG findings and syncope. However, an electrophysiological study performed during cannabis consumption and after sufficiently long withdrawal period could help explaining how cannabinoids could impair hearth conduction system [13]. Even if it was clearly explained that pacemaker implantation is linked with lifelong potential complications and could have not been a resolutive intervention, she decided not to try to abandon cannabis abuse, even under medical supervision, in favor of pacemaker implantation.

24 months follow up was completely free from syncopal episodes. However, due to relatively low but higher than expected ventricular pacing burden of 14%, we changed the pacemaker settings to single-chamber atrial pacing with back-up ventricular pacing (AAI- > DDD) with *managed ventricular pacing* algorithm (MVP™; Medtronic, Minneapolis, MN, USA) to reduce right ventricular pacing. Such algorithm should further reduce ventricular pacing performing in AAI mode when AV block is not present and by automatically switch to DDD mode when AV block is detected. Moreover, we performed an exercise stress test that did not show any evidence of chronotropic dysfunction, so we removed rate response algorithm to further decrease unnecessary ventricular pacing.

Echocardiogram findings after two years were normal. Still, the patient complaint about sporadic pre-syncopal episodes, which were never linked to specific intracardiac electrogram findings. Even if the patient declared that she stopped smoking cannabis during pregnancy, we cannot completely exclude persistent consumption since we did not perform toxicological tests during follow up.

Considering the young age of our patient, extended follow up will be needed to better understand the evolution of a potential underlying conduction disorder and to refine pacemaker settings to obtain the lowest ventricular pacing burden possible to minimize the risk of prolonged right ventricular pacing.

More in general, synergic and potentially lethal effects when cannabis is consumed with other drugs (e.g., cocaine) should be considered by all clinicians [14]. It could be useful to evaluate any preclinical alterations of cardiac function as observed with other drugs of abuse and doping agents [15]. Furthermore, there is a growing body of evidence pointing to the co-occurrence of cannabis use and depression [16]: as regards cardiovascular field, these patients may have a higher incidence of Takotsubo Syndrome with possible acute myocardial dysfunction [17].

Lastly, in cases of recorded asystole from hypervagotonia in young people, the hypothesis of heavy chronic cannabis use must be indagated, with toxicological analysis of hair samples, because conventional drug testing (urine) is not adequate to identify the chronic use [18]. Results obtained in hair samples can demonstrate a long heavy chronic use in these patients and this could explain asystole episodes from hypervagotonia.

In conclusion, cannabis cardiovascular effects are not well known; among these we find ischemic episodes, tachyarrhythmias, symptomatic sinus bradycardia, sinus arrest, ventricular asystole and possibly death. In the light of the poor literature, we believe that cannabis may produce opposite adverse effects depending on the duration of the habit. Acute administration increases sympathetic tone and reduces parasympathetic tone; conversely, with chronic intake an opposite effect is observed (both should be confirmed with toxicological analysis): repetitive dosing decreases sympathetic activity and increases parasympathetic activity. With this paper, we aim to promote drug consumption investigation in young patients presenting with both brady and tachydysrhythmias. Physicians should be aware of the effects that cannabis produces upon the cardiovascular system: this could avoid expensive, prolonged hospitalizations and needless diagnostic tests.

Because relatively rare, cannabis adverse effects have received surprisingly little clinical attention and its consumption is perceived as relatively safe from general population. However, its growing consumption may be responsible for an increase of these events that could endanger health of individual users and of the society in its entirety.

## Data Availability

All data generated or analyzed during this study are included in this published article.

## Abbreviations

MI: Myocardial infarction
ILR: Implantable loop recorder
BP: Blood pressure
HR: Heart rate
O2: Oxygen
ECG: Electrocardiogram
CBD: Cannabidiol
THC: Tetrahydrocannabinol
EEG: Electroencephalogram
AVB: Atrio-ventricular block
DDD: Dual-chamber pacing mode pacemaker
AAI: Single-chamber atrial pacing mode pacemaker
